# Survival outcomes in locally advanced dMMR rectal cancer: surgery plus adjunctive treatment vs. surgery alone

**DOI:** 10.1186/s12885-023-11525-7

**Published:** 2023-10-20

**Authors:** Kemin Ni, Yixiang Zhan, Zhaoce Liu, Zhen Yuan, Shuyuan Wang, Xuan-zhu Zhao, Hangyu Ping, Yaohong Liu, Wanting Wang, Suying Yan, Ran Xin, Qiurong Han, Qinghuai Zhang, Guoxun Li, Xipeng Zhang, Guihua Wang, Zili Zhang, Hong Ma, Chunze Zhang

**Affiliations:** 1 School of Medicine, Nankai University, Tianjin, 300110 China; 2 Department of Colorectal Surgery, Tianjin Union Medical Center, Tianjin, 300121 China; 3 School of Integrative Medicine, Tianjin University of Traditional Chinese Medicine, Tianjin, 300193 China; 4 The Institute of Translational Medicine, Tianjin Union Medical Center of Nankai University, Tianjin, 300121 China; 5 Tianjin Institute of Coloproctology, Tianjin, China; 6 Tongji Hospital, Tongji Medical College, Huazhong University of Science and Technology, Wuhan, 430030 China; 7 The Third Central Clinical College of Tianjin Medical University, Tianjin, 300171 China; 8 Nursing Department, Tianjin Union Medical Center, Tianjin, 300121 China

**Keywords:** Rectal Cancer, dMMR, Treatment, Surgery, Prognosis

## Abstract

**Background:**

Recent studies have shown that deficient mismatch repair (dMMR) rectal cancer may be related to treatment resistance, resulting in a worse prognosis than proficient MMR (pMMR) rectal cancer. The purpose of this study was to explore whether surgery plus other treatments (radiotherapy and chemotherapy) can bring more benefits to these patients than surgery alone.

**Methods:**

A retrospective study of 168 patients with rectal adenocarcinoma who underwent total mesorectal excision was conducted using immunohistochemical methods to determine MMR status and a propensity score matching model to minimize potential confounding factors between subgroups of patients with different treatment regimens. Kaplan–Meier analysis, log-rank tests, and Cox regression models were used to assess overall survival (OS) and disease-free survival (DFS) in patient subgroups.

**Results:**

Only 6.9% (*n* = 168) of patients in the total cohort had dMMR rectal adenocarcinoma, and the most common cause of dMMR was a PMS2 deletion (103, 61.3%). The median DFS of the surgery alone group was 45.7 months (IQR, 40.9 to 77.8), and the median DFS of the surgery plus other treatment group was 43.9 months (IQR, 14.2 to 80.1). The surgery alone group was superior to the surgery plus other treatment group (HR, 0.16; 95% CI, 0.07 to 0.38; *p* = 0.005). There was no significant difference in OS (45.8 (IQR, 41.0 to 79.8) vs. 45.9 (IQR, 38.5 to 80.3)) between the two groups (HR, 0.57; 95% CI, 0.23 to 1.40; *p* = 0.263).

**Conclusions:**

For patients with locally advanced dMMR rectal adenocarcinoma, compared with surgery alone, surgery plus other treatment options (radiotherapy and chemotherapy) do not grant long-term survival benefits but rather shorten DFS.

**Supplementary Information:**

The online version contains supplementary material available at 10.1186/s12885-023-11525-7.

## Background

Colorectal cancer (CRC) is the third most common malignant tumor and the second most common cause of death globally. According to the GLOBOCAN project of the WHO Cancer Research Center, the number of new CRC cases worldwide in 2020 was approximately 1.89 million, and the number of deaths was approximately 916 thousand. Rectal cancer accounts for approximately 38.8% of all newly diagnosed CRC cases [1], and the majority of patients present with locoregional disease [2]. Although the current options for the treatment of rectal cancer vary, with the advent of precision medicine, an increasing number of doctors are carrying out individualized treatment plans according to the characteristics of a patient's disease, including clinical stage, pathological classification, biomarkers, and so on, to achieve better treatment results and avoid damage caused by overtreatment. Deficient mismatch repair (dMMR), which results in microsatellite instability (MSI), is an important molecular biological marker of CRC.

Primary proteins in the DNA Mismatch Repair (MMR) system—including MLH1, MSH2, MSH3, MSH6, and PMS2—are responsible for rectifying errors that occur during DNA replication [3]. When these proteins are deficient, replication errors uncorrected, leading to the accumulation of DNA mutations. This results in an elevated rate of natural mutations. This phenomenon is notably prevalent in microsatellite repeat regions that are prone to replication errors [4]. Such deficiencies not only lead to MSI but also elevate the risk of colorectal carcinogenesis [5]. Because the detection of MSI/dMMR is helpful to guide treatment [6], it is recommended to detect MSI/dMMR in all CRC patients [7].

Although conflicting evidence exists in studies [8–10] concerning the prognostic value of dMMR status in rectal cancer patients, the majority of academic opinion still supports a more favorable prognosis for patients identified with dMMR status. Research conducted by Rosa N et al. [11] found that patients with dMMR rectal cancer displayed superior prognostic outcomes and pathological responses under comprehensive treatment strategies, including individualized surgical approaches. This study, which incorporated the largest available quantity of clinical data related to dMMR rectal cancer patients at that time, possesses significant persuasive power. The National Comprehensive Cancer (NCCN) guidelines recommend neoadjuvant chemoradiotherapy (NCRT) and surgery plus adjuvant chemotherapy for patients with stage II/III rectal cancer [7]. However, different treatment options may be needed for patients with dMMR rectal cancer. For example, a recent study found that an appropriate reduction in treatment measures increased the therapeutic benefits of these patients [12–14].

However, patients with dMMR rectal cancer are relatively rare, so there is a lack of research on the effects of different treatment strategies on their prognosis. The purpose of this study was to explore whether surgery plus other treatments can provide more benefits than surgery alone and subsequently identify the treatment strategy that is most suitable for this particular population to guide treatment selection for future patients.

## Patients and methods

### Study design and participants

**Fig. 1:**
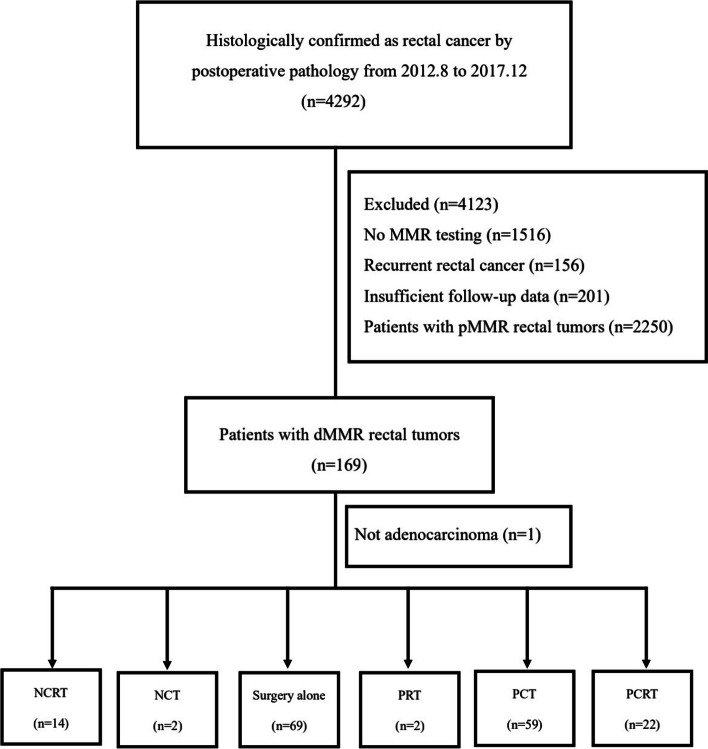
Flowchart depicting the process of patient inclusion. MMR mismatch repair, pMMR proficient mismatch repair, dMMR deficient mismatch repair, NCRT neoadjuvant chemoradiotherapy and surgery plus adjuvant chemotherapy, NCT Neoadjuvant chemotherapy, PRT surgery combined with postoperative radiotherapy, PCT surgery plus postoperative chemotherapy, PCRT surgery plus postoperative chemoradiotherapy

This retrospective multicenter study included patients with rectal cancer who underwent radical rectal cancer surgery at Tianjin Union Medical Center, Tianjin Third Central Hospital, and Tongji Hospital from August 2012 to December 2017. We reviewed the patients' clinical files, treatment plans, and surgical, radiological, and pathological reports. Highlights of the reports included age, sex, clinical and radiological tumor stage at diagnosis, pathological features, specific regimens of neoadjuvant and adjuvant therapy, local and/or distant recurrence, and death. Relevant medical and family history of the patient was obtained through an interview with the patient or his next of kin. Of these, cases in which the diagnosis of rectal cancer was not their first and primary malignancy were excluded, and patients with a history of inflammatory bowel disease or known familial adenomatous polyposis were also excluded from this study. Initially, 4292 patients with rectal cancer were included. As recommended, all patients were assessed for T and N status by magnetic resonance imaging or ultrasound (echo-endoscopy) prior to initial treatment. If different staging was reported for the two assessment modalities, patients were assigned to the worse one. Staging was defined according to the American Joint Committee on Cancer 7th edition clinical staging of rectal cancer. All patients had previously consented to have their data used in the retrospective study. The study was approved by the Ethics Committee of Tianjin Union Medical Center (Ethical Approval Number: 2021-B16) and followed the reporting recommendations of the tumor marker study (REMARK) guidelines [15]. Only 168 patients with dMMR status confirmed by tissue specimens were finally included. A schematic representation of this selection process is depicted in Fig. 1.

### Multimodality treatments for rectal cancer

All patients underwent purposeful total mesorectal excision (TME). The NCRT group was treated with continuous 5-FU titration (225 mg/(m^2^·d), 5 days per week) during radiotherapy, and TME was performed 4–6 weeks after completion of preoperative chemoradiotherapy. In contrast, the NCT group underwent preoperative chemotherapy only, and the chemotherapy regimen was identical to that of the NCRT group. The PCRT group was started with mFOLFOX6 regimen chemotherapy within 3 weeks after surgery, and then continuous radiation therapy was started at week 4. The PCT group was started with mFOLFOX6 regimen chemotherapy within 3 weeks after surgery only, while the PRT group was started with continuous radiotherapy without chemotherapy. The specific regimen of radiotherapy was 1.8 to 2.0 Gy per day from Monday to Friday for a total of 23 to 25 doses over 5 to 6 weeks (total dose 46.0 to 50.4 Gy). Photons at a minimal energy of 6-MV were delivered to the primary tumor and rectal mesenteric lymph nodes, presacral lymph nodes, and internal iliac lymph nodes via a three-field or four-field box technique. The treatment plan for all patients was made after discussion by the multidisciplinary oncology committee based on Chinese treatment guidelines [16] and in combination with the patient's own willingness.

### MMR status determination and analysis

To evaluate the MMR status, the expression of MMR genes/proteins (MLH1, PMS2, MSH6, and MSH2) was analyzed using immunohistochemical (IHC) staining. ZSGB-Bio Solutions SPlink Detection Kits (Zhongshan Jinqiao Biotechnology, Beijing, China) and an automated IHC/ISH slide staining instrument (Benchmark, Roche, Arizona, USA) were used to stain formalin-fixed, paraffin-embedded, 5-μm sections according to the manufacturer's instructions. Staining was conducted with diagnostic antibodies against MLH1 (clone OTI4H4; Zhongshan Jinqiao Biotechnology, Beijing, China, 1:40), MSH2 (clone RED2; Zhongshan Jinqiao Biotechnology, Beijing, China, 1:200), MSH6 (clone UMAB258; Zhongshan Jinqiao Biotechnology, Beijing, China, 1:200) and PMS2 (clone EP51; Zhongshan Jinqiao Biotechnology, Beijing, China, 1:40). MMR proteins are localized in the nucleus, and loss of their expression is defined as the absence of nuclear staining in tumor cells. In contrast, nuclear staining occurs in normal epithelial cells, infiltrating lymphocytes and stromal cells around the tumor. If the expression of ≥ 1 MMR protein was lost, the patient was allocated to the dMMR group; otherwise, the patient was allocated to the pMMR group.

### Oncologic follow-up for rectal cancer

All patients were followed until last contact or death. The vital status and cause of death were obtained from medical records or death certificates. The surveillance included a physical examination with an emphasis on the digital rectal examination; blood CEA levels; liver ultrasonography; chest, abdominal, and pelvic CT/MRI; and colonoscopy [16].

### Propensity score matching

A propensity score model was used to match potential bias in confounding covariates for patients in the surgery-alone treatment group and the surgery-plus-other-treatment group. The propensity score model was performed by matching the potential confounding clinicopathological factors, including age (≤ 60 y vs. > 60 y) at diagnosis, sex (male vs. female), clinical stage (II vs. III), histologic grade (well, moderately, poorly differentiated), and neurovascular invasion (negative vs. positive). The surgery-alone treatment group and the surgery-plus-other-treatment group were matched at a 1:2 ratio using a greedy, nearest neighbor matching algorithm with no replacement. In propensity score matching methodology, the choice of matching tolerance is crucial. A larger matching tolerance facilitates a greater number of successful individual matches, thus expanding the size of the matched cohort. However, this often comes at the expense of deteriorating between-group balance. On the other hand, a smaller matching tolerance can improve between-group balance but may reduce the rate of successful matching, leading to a smaller matched sample size. For optimal matching tolerance, we have chosen 20% of the standard deviation of the propensity scores for both groups, which is utilized as the logit for propensity score calculations. Differences in patient characteristics between the propensity score-matched subgroups were assessed using p-values.

### Statistical analysis

Categorical data are described as the number and percentage, and continuous data are described as the median and interquartile range (IQR). Overall survival (OS) was defined as the interval between the initiation of therapy and either death from any cause or the date of the last follow-up for surviving patients. Disease-free survival (DFS) was defined as the time from the first day of therapy to the date of cancer recurrence, death from any cause, or the last follow-up for patients with no evidence of recurrence. Survival curves were generated by the Kaplan–Meier method, and univariable analysis between these subgroups was performed by using the log-rank test. Hazard ratios and their associated 95% confidence intervals were calculated with the use of a Cox proportional hazards model. *P*-values < 0.05 were considered statistically significant, and all reported P-values were 2-sided. All analyses were performed using IBM SPSS version 26.

## Results

### Patient characteristics

Patient characteristics are summarized in Table 1. Of the 2419 patients with primary rectal cancer who had MMR testing and complete information, 168 rectal adenocarcinoma tumors (6.9%) were classified as dMMR. The median age at the time of diagnosis was 60 years (IQR: 53 to 68), and 98 of 168 patients were male (58.3%).

**Table 1:** Patient and treatment characteristics

Characteristics	No. of Patients (%)
Age at rectal cancer diagnosis, median (IQR),years	60 (53–68)
Sex	
Male	98 (58.3)
Female	79 (41.7)
Clinical stage of rectal cancer at diagnosis	
cT1-T2N0	26 (15.5)
cT3-T4N0	74 (44.0)
cTanyNpositive	60 (35.7)
cTanyNanyMpositive	8 (4.8)
Tumor histologic grade	
Well differentiated	6 (3.6)
Moderately differentiated	150 (89.3)
Poorly differentiated	12 (7.1)
Mucinous tumor	46 (28.0)
Tumor distance from anal verge, median (IQR), cm	6 (4–10)
≤ 5 cm	65 (38.7)
> 5 and ≤ 10 cm	60 (35.7)
> 10	43 (25.6)
Resection margin status	
Positive	3 (1.8)
Negative	165 (98.2)
Neurovascular invasion	
Yes	17 (10.1)
No	151 (89.9)
MMR status (-)	
MLH1	56 (33.3)
PMS2	103 (61.3)
MSH2	36 (21.4)
MSH6	36 (21.4)
Treatment	
NCRT	14 (8.3)
NCT	2 (1.2)
Surgery alone	69 (41.1)
PRT	2 (1.2)
PCT	59 (35.1)
PCRT	22 (13.1)
Surgical procedure	
Dixon	99 (58.9)
Miles	43 (25.6)
Hartmann	20 (11.9)
Others	6 (3.6)

*Abbreviations*: *IQR* interquartile range, *NCRT* neoadjuvant chemoradiotherapy and surgery plus adjuvant chemotherapy, *NCT* Neoadjuvant chemotherapy, *PRT* surgery combined with postoperative radiotherapy, *PCT* surgery plus postoperative chemotherapy, *PCRT* surgery plus postoperative chemoradiotherapy

The pathological diagnoses were as follows: poorly differentiated adenocarcinoma in 12 patients (7.1%), moderately differentiated adenocarcinoma in 110 patients (65.5%), well differentiated adenocarcinoma in 6 patients (3.6%), and mucous adenocarcinoma in 40 patients (23.8%). The median tumor distance from the anal verge was 6 cm (IQR, 4 to 10 cm). At the time of diagnosis, 134 patients (79.8%) had locally advanced rectal cancer (stages II and III), while only 26 (15.5%) patients were in stage I. Eight patients (4.7%) had stage IV disease involving the liver (*n* = 6) and lung (*n* = 2).

The treatment strategies are shown in Table 1. In total, 14 patients received NCRT, 2 patients received neoadjuvant chemotherapy (NCT), 69 patients received surgery alone, 2 patients received surgery combined with postoperative radiotherapy (PRT), 59 patients received surgery plus postoperative chemotherapy (PCT) and 22 patients received surgery plus postoperative chemoradiotherapy (PCRT).

At 1:2 propensity score matching, 35 patients treated with surgery alone were matched with 56 patients treated with surgery plus other treatments, as shown in Table 2, and the standardized differences of the covariates included in both subgroups of patients after propensity score matching were less than 0.1 (Supplementary Table 1), indicating a well-balanced covariate distribution.

**Table 2:** Baseline characteristics of patients with different treatment regimens before and after matching

	Before Matching			After Matching (1:4)		
**Characteristics**	surgery-alone, *N* = 39	surgery-plus-other-treatment *N* = 95	***P***	surgery-alone, *N* = 35	surgery-plus-other-treatment *N* = 56	***P***
Sex (%)			0.356			1.000
Male	20 (51.3)	57 (60.0)		20 (57.1)	32 (57.1)	
Female	19 (48.7)	38 (40.0)		15 (42.9)	24 (42.9)	
Age (year, %)			0.001			0.303
≤ 60	13 (33.3)	62 (65.3)		13 (37.1)	27 (48.2)	
> 60	26 (66.7)	33 (34.7)		22 (62.9)	29 (51.8)	
Clinical stage (%)			0.004			0.721
II	29 (74.4)	45 (47.4)		25 (71.4)	38 (67.9)	
III	10 (25.6)	50 (52.6)		10 (28.6)	18 (32.1)	
Tumor histologic grade (%)			0.848			0.842
Well	1 (2.6)	3 (3.1)		1 (2.8)	0 (0)	
Moderately	35 (89.7)	83 (87.4)		31 (88.6)	52 (92.9)	
Poorly	3 (7.7)	9 (9.5)		3 (8.6)	4 (7.1)	
Neurovascular invasion (%)			0.890			0.550
Yes	4 (10.3)	9 (9.5)		3 (8.6)	3 (5.4)	
No	35 (89.7)	86 (90.5)		32 (91.4)	53 (94.6)	

Clinicopathological differences between the surgery-alone treatment group and the surgery-plus-other-treatment group were compared with the Mann–Whitney U test for continuous variables and χ2 test (or Fisher’s exact test, if appropriate) for categorical data

### MMR status

dMMR was most frequently due to defective PMS2 (103 patients, 61.3%), followed by MLH1 (56 patients, 33.3%), MSH2 (36 patients, 21.4%), and MSH6 (36 patients, 21.4%). Simultaneous loss of the expression of MLH1 and PMS2 was observed in 40 patients (23.8%), and the coloss of MSH2 and MSH6 was observed in 23 patients (13.7%).

### Treatment and long-term prognosis

**Fig. 2:**
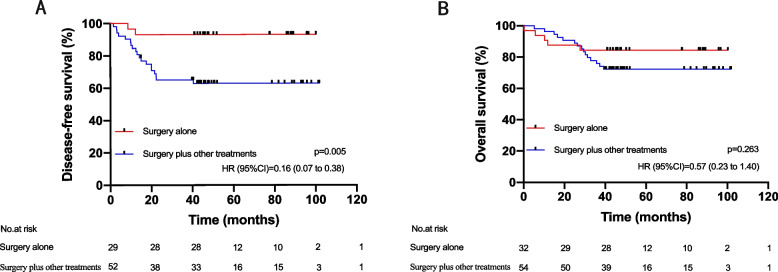
**A** Disease-free survival (DFS) of LARC patients treated with surgery alone and surgery plus other treatments. **B** Overall survival (OS) of LARC patients treated with surgery alone and surgery plus other treatments. LARC, locally advanced rectal cancer (stages II and III); HR, hazard ratio; CI, confidence interval

**Fig. 3:**
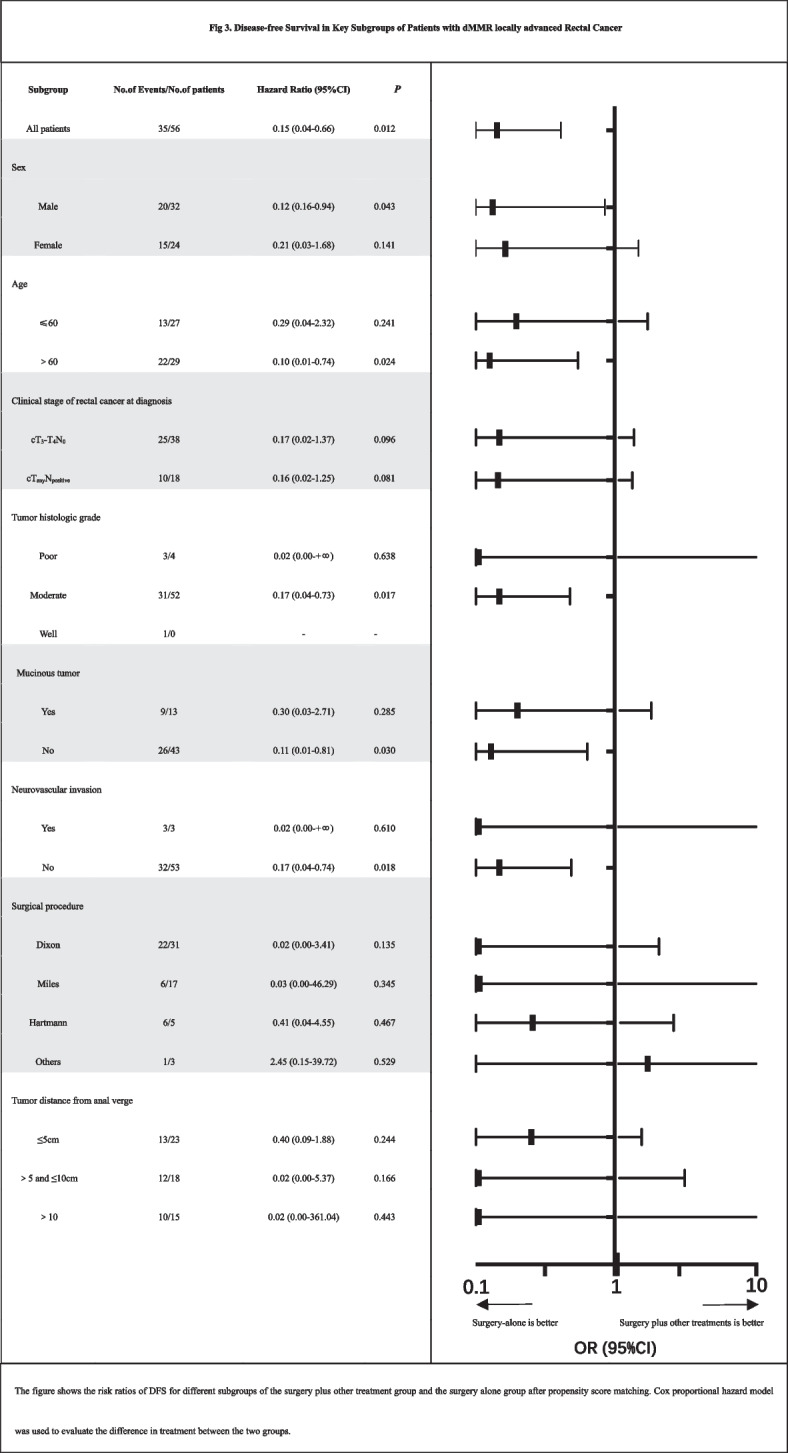
Disease-free Survival in Key Subgroups of Patients with dMMR locally advanced Rectal Cancer. The figure shows the risk ratios of DFS for different subgroups of the surgery plus other treatment group and the surgery alone group after propensity score matching. A Cox proportional hazard model was used to evaluate the difference in treatment between the two groups

**Fig. 4:**
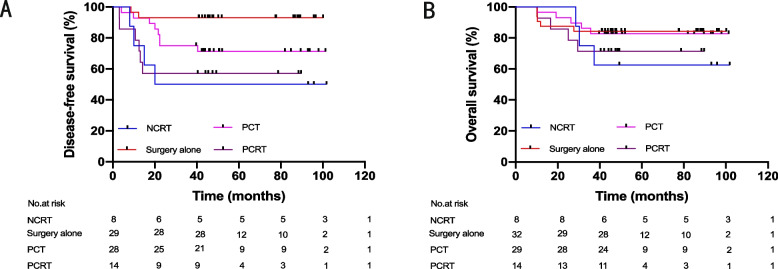
**A** Disease-free survival (DFS) of LARC patients with different treatments. **B** Overall survival (OS) of LARC patients with different treatments. LARC locally advanced rectal cancer (stages II and III), NCRT neoadjuvant chemoradiotherapy and surgery plus adjuvant chemotherapy, PCT surgery plus postoperative chemotherapy, PCRT surgery plus postoperative chemoradiotherapy

The median follow-up period of this cohort was 46.0 months (IQR, 39.5 ~ 80.3). The 3-year OS and DFS rates were 81.0% and 79.2%, respectively. The three-year OS rates of stage I and IV patients were 100% and 12.5%, respectively. In contrast, the prognosis of locally advanced rectal cancer (stages II and III) varied according to the treatment regimen, in which the median DFS of the surgery alone group was 45.7 months (IQR, 40.9 to 77.8), and the median DFS of the surgery plus other treatment group was 43.9 months (IQR, 14.2 to 80.1). The OS for the two treatment groups was 45.8 (IQR, 41.0 to 79.8) vs. 45.9 (IQR, 38.5 to 80.3). Analysis of the cohort after 1:2 propensity score matching revealed that DFS was better in the surgery alone treatment group than in the surgery plus other treatment group (HR, 0.16; 95% CI, 0.07 to 0.38; *p* = 0.005, Fig. 2A). Based on the analysis of the risk ratio of DFS with different treatment options in each subgroup, it was found that the DFS rate of the surgery alone group was always better than that of the surgery plus other treatment group (Fig. 3). There was no significant difference in OS between the two groups (HR, 0.57; 95% CI, 0.23 to 1.40; *p* = 0.263, Fig. 2B). Additionally, it is widely acknowledged that mucinous adenocarcinomas are considered grade 3 tumors and represent a special subtype of colorectal carcinoma. They also constitute a significant proportion among dMMR colorectal cancer patients. Therefore, we conducted a separate analysis excluding all patients with mucinous adenocarcinomas. The results remained consistent with our initial conclusions (Supplementary Fig. 1). Furthermore, to validate our findings, we employed an alternative approach by incorporating 'mucinous adenocarcinoma' as a predictive variable in propensity score matching. The conclusions derived from this modified analysis were also congruent with our original findings (Supplementary Fig. 2). After subdividing patients according to the treatment plan and then performing pairwise comparisons of DFS and OS (Fig. 4, Supplementary Table 2), the conclusion was consistent; that is, there was no significant difference in OS between the groups, and treatment other than surgery did not bring survival benefits to patients with dMMR locally advanced rectal cancer. Compared with surgery alone, NCRT (HR, 0.12; 95% CI, 0.01 to 0.90; *p* = 0.003), PCT (HR, 0.22; 95% CI, 0.06 to 0.77; *p* = 0.038), and PCRT (HR, 0.14; 95% CI, 0.03 to 0.63; *p* = 0.004) were associated with worse DFS. The other two treatments (NCT and PRT) were not analyzed because of too few data.

## Discussion

dMMR/MSI rectal tumors are biologically and clinically distinct [3]; however, the effect of the dMMR/MSI status on the treatment of rectal adenocarcinoma is not clear, and there is a lack of research on the effects of different treatment strategies on the prognosis of rectal adenocarcinoma because of the scarcity of such patients. To our knowledge, our study has the largest number of patients with dMMR rectal adenocarcinoma currently included in Asia and used a propensity score model to balance the baseline between subgroups. After comparing the OS and DFS of patients treated with different strategies, we found that patients with locally advanced dMMR rectal adenocarcinoma may not benefit from radiotherapy and chemotherapy. This conclusion can provide guidance for current treatment explorations.

Our study shows that for patients with locally advanced (stages II/III) dMMR rectal adenocarcinoma, compared with surgery alone, surgery plus radiotherapy and chemotherapy not only does not benefit long-term survival but also worsens DFS. A recent study by Shu-Biao et al. [12] showed that the DFS rate of dMMR patients receiving neoadjuvant chemoradiotherapy was significantly lower than that of patients receiving neoadjuvant chemotherapy. This is consistent with our study. In addition, Sargent et al. [17] compared the prognosis of stage II and III dMMR colon cancer patients treated with surgery alone and surgery plus PCT. The authors concluded that the prognosis was better in the surgery alone group. Therefore, we speculate that when patients with dMMR tumors are treated with radiotherapy and chemotherapy, the damage caused by the treatment may outweigh the benefits. A previous study evaluated a group of patients from the National Cancer Database with locally advanced rectal cancer who received chemoradiotherapy prior to surgical resection. This group included 5086 patients, with 4450 patients having pMMR tumors and 636 patients having dMMR tumors. Statistical analysis showed that dMMR was correlated with a decrease in the pathologic complete response (pCR) rate, suggesting that dMMR tumors are resistant to chemotherapy and radiotherapy [18]. Recent studies by Cercek et al. have also reached the same conclusion, further supporting our speculation [14].

Historically, dMMR/MSI has been a predictive marker indicating that patients with colorectal tumors have limited benefits from fluorouracil treatment [17, 19]. In our cohort, the DFS rate of patients with dMMR tumors treated with postoperative chemotherapy was significantly worse than that of patients treated with surgery alone (*P* = 0.038). In vitro studies have shown that dMMR is the cause of tumor resistance to fluorouracil [20]. The MMR component is necessary for many DNA damage agents to induce apoptosis. The possible mechanism is that fluorouracil and uracil are similar in structure and can be synthesized into fluorouracil deoxynucleotides in cells. Fluorouracil deoxynucleotides are raw materials that partially replace thymine deoxynucleotides as raw materials for DNA synthesis, while the MMR complex can specifically recognize and bind to fluorouracil-modified DNA to induce cell apoptosis. In the absence of MMR, tumor cells become resistant to fluorouracil-based chemotherapy [21]. There is also a hypothesis that dMMR tumor lymphocyte infiltration causes an antitumor immune response [22] and that this immune response may be eliminated by the immunosuppressive effect of chemotherapy. Therefore, chemotherapy cannot benefit patients with dMMR tumors.

In locally advanced rectal cancer, radiotherapy is usually used to improve local control [23]. To date, clinical trials have not confirmed that radiotherapy can improve DFS and OS [23, 24]. In our cohort, too few patients received radiotherapy plus surgery (2 patients), making it difficult to perform a direct comparison, but compared with the postoperative plus chemotherapy group a, the addition of radiotherapy did not significantly improve OS (*P* = 0.425) or DFS (*P* = 0.317). Shin et al. proposed that radiotherapy delivers ionizing radiation to target cells to induce their death by causing various damage to their genomes. Among them, a double-strand break (DSB) on chromosomal DNA is considered to be the most fatal form of damage, where both strands of the DNA helix are severed. In response, a highly complex and incompletely understood network of intracellular molecular pathways, collectively referred to as the DNA damage response (DDR), is triggered [25]. There is increasing evidence that DNA MMR proteins may affect and/or directly participate in radiation-induced DDR after a DSB. In other words, dMMR tumors may be more sensitive to radiotherapy. Animal experiments have proven that dMMR tumors are more sensitive to radiotherapy than pMMR tumors [26–28]. However, clinical radiotherapy does not benefit patients with dMMR tumors [12, 14] but rather results in radiotherapy resistance, and the mechanism is not clear. Some studies have shown the accelerated growth of intestinal tumors after radiation exposure in MLH1-knockout mice [29]. This finding suggests an increased risk of secondary cancer in dMMR tumors treated with radiation. In clinical practice, it has also been found that radiotherapy increases the probability of secondary tumors [30]. Therefore, although patients with dMMR tumors may theoretically be more sensitive to radiotherapy, the side damage caused by radiotherapy may exceed the benefits of treatment.

In addition, our data confirm once again that the proportion of dMMR rectal cancer (6.9%) is lower than that of CRC as a whole (10%-15%) [31, 32]. We also found that the proportion of Chinese patients with dMMR rectal cancer was surprisingly consistent with that in a large cohort study [12]. Additionally, we found that the most common mismatch repair protein deletion in the Chinese population was PMS2. Zeng et al. [33] recently reported that patients with dMMR tumors accounted for 11.8% of the CRC population, while those with PMS2 deletion accounted for 8.3% of the total CRC population, meaning that 70.3% of the dMMR tumor patients in their cohort had PMS2 protein deletion, which is consistent with the presence of PMS2 deletion in 61.3% of dMMR rectal cancer patients in our cohort. However, this is at variance with previously reported data from Western countries. In previous studies, MSH2 or MLH1 deletions were often the most common [11, 34]. Although there is no clear explanation for this phenomenon, it can be speculated that it may be caused by differences in ethnicity, socioeconomic status, lifestyle, eating habits, or environment.

Although our findings are thought-provoking and provide strong clinical data to support the conclusions of previous studies [14], these results are still subject to the selection bias inherent in large retrospective studies. For example, a significant proportion of individuals (approximately 35%) would be clinically excluded because they had no MMR testing. Furthermore, despite our best efforts to include as many dMMR rectal cancer patients as possible, the rarity of this patient population inevitably constrained the total number of inclusions. Consequently, the limited sample size may affect the overall robustness of our findings, and the persuasive power of our results may be somewhat diminished. The rarity of dMMR rectal cancer also complicates the execution of prospective randomized studies, emphasizing the challenges inherent in obtaining larger sample sizes for this specific subgroup. Furthermore, in the Chinese context, as the time between diagnosis and treatment initiation for our patients was notably short, we only recorded the time of tumor diagnosis and considered it synonymous with treatment initiation, an approach that lacks rigor. Despite the rigor of our statistical methods, including propensity score matching, our conclusions should be interpreted with caution. We acknowledge the absence of double-validation for dMMR status, as polymerase chain reaction (PCR) for microsatellite loci amplification was not performed due to the retrospective study design. Additionally, in China—a developing country where the importance of MMR status determination has recently gained recognition—our study relied on post-surgical specimens for MMR status, as our data collection began in 2013 and pre-surgical tissue samples were often unavailable. However, literature suggests [35] that MMR status is generally stable between pre- and post-surgical specimens, except in patients subjected to neoadjuvant chemoradiotherapy (NCRT). Since a large proportion of patients in our study did not undergo NCRT, this lends some credence to the clinical implications of our findings. Given these factors, our results emphasize the need for future studies with more robust, prospective designs for unequivocal conclusions. It is imperative at this juncture to also highlight the rapidly evolving landscape of immunotherapies. Emerging literature consistently demonstrates the efficacy of checkpoint inhibitors such as pembrolizumab and nivolumab in dMMR tumors. These therapies offer promising clinical outcomes with fewer treatment-related adverse events [36–38]. Particularly in tumors with high levels of microsatellite instability (MSI-H), a characteristic feature of dMMR cancers [39], immunotherapies hold the potential to establish a new standard of care. With long-term benefits like durable response rates and improved overall survival, immunotherapies may soon serve as a viable alternative to, or even replace, traditional treatments such as surgery, chemotherapy, and radiation [40]. Given these advancements, future treatment protocols for dMMR rectal adenocarcinoma might increasingly incorporate immunotherapy, either as an adjunct to surgery or as an independent treatment modality.

## Conclusions

In summary, our study underscores that patients with dMMR rectal adenocarcinoma do not obtain significant benefit from chemoradiotherapy. Specifically, for those with locally advanced dMMR rectal adenocarcinoma, undergoing surgery alone resulted in no worse long-term survival as compared to combining surgery with chemoradiotherapy, and even showed superior disease-free survival rates. Consequently, we strongly recommend a more conservative treatment approach for these patients. However, it's important to note the rapidly advancing field of immunotherapies, which could change treatment protocols for dMMR rectal adenocarcinoma in the near future. Post-surgery, some patients may require no additional treatments unless disease progression is observed, or surgery could be combined with immunotherapies. These emerging approaches, nonetheless, necessitate further validation through prospective, multicenter, randomized, controlled clinical trials.

### Supplementary Information

**Additional file 1:** **Supplementary Table 1.** Detailed Balance.

**Additional file 2:** **Supplementary Table 2.** Abbreviations: DFS, disease-free survival; OS, overall survival; NCRT, neoadjuvant chemoradiotherapy and surgery plus adjuvant chemotherapy; PCT, surgery plus postoperative chemotherapy; PCRT, surgery plus postoperative chemoradiotherapy.

**Additional file 3:** **Supplementary Fig. 1. **(A) Disease-free survival (DFS) of LARC patients treated with surgery-alone and other treatments. (B) Overall survival (OS) of LARC patients treated with surgery alone and other treatments. LARC, locally advanced rectal cancer (stages II and III); HR, hazard ratio; CI, confidence interval.

**Additional file 4:** **Supplementary Fig. 2. **(A) Disease-free survival (DFS) of LARC patients treated with surgery-alone and other treatments.(B) Overall survival (OS) of LARC patients treated with surgery alone and other treatments. LARC, locally advanced rectal cancer (stages II and III); HR, hazard ratio; CI, confidence interval.

## Data Availability

The original contributions presented in the study are included in the article/Supplementary Materials. Further inquiries can be directed to the corresponding author.

## Abbreviations

dMMR: Deficient mismatch repair
OS: Overall survival
DFS: Disease-free survival
pMMR: Proficient mismatch repair
CRC: Colorectal cancer
MMR: Mismatch repair
IHC: Immunohistochemistry
pCR: Pathologic complete response
IQR: Interquartile range
NCRT: Neoadjuvant chemoradiotherapy
NCT: Neoadjuvant chemotherapy
PRT: Surgery combined with postoperative radiotherapy
PCT: Surgery plus postoperative chemotherapy
PCRT: Surgery plus postoperative chemoradiotherapy
