# Supplementary material for: Survival outcomes in locally advanced dMMR rectal cancer: surgery plus adjunctive treatment vs. surgery alone
Source: BMC Cancer. 2023 Oct 20;23:1013. doi: 10.1186/s12885-023-11525-7 (PMC10588073; doi:10.1186/s12885-023-11525-7)
Supplement: Supplementary file 1 — Additional file 1: Supplementary Table 1. Detailed Balance. [file 12885_2023_11525_MOESM1_ESM.docx]

| Supplementary Table1  **Detailed Balance** | | | | | | |
| --- | --- | --- | --- | --- | --- | --- |
| Subsamples Covariates | Means Treated | | Means Control | | Std. Mean Diff. | |
|  | Before | After | Before | After | Before | After |
| Sex | 0.5128 | 0.5714 | 0.6 | 0.5714 | -0.1744 | 0 |
| Age at Diagnosis | 1.6667 | 1.6286 | 1.3474 | 1.6143 | 0.6686 | 0.0299 |
| Clinical Stage | 2.2564 | 2.2857 | 2.5263 | 2.2714 | -0.6102 | 0.0323 |
| Histologic Grade | 1.9487 | 1.9429 | 1.9368 | 1.9429 | 0.0371 | 0 |
| Neurovascular invasion | 0.1026 | 0.0857 | 0.0947 | 0.0714 | 0.0258 | 0.0471 |
