# Supplementary material for: Survival outcomes in locally advanced dMMR rectal cancer: surgery plus adjunctive treatment vs. surgery alone
Source: BMC Cancer. 2023 Oct 20;23:1013. doi: 10.1186/s12885-023-11525-7 (PMC10588073; doi:10.1186/s12885-023-11525-7)
Supplement: Supplementary file 2 — Additional file 2: Supplementary Table 2. Abbreviations: DFS, disease-free survival; OS, overall survival; NCRT, neoadjuvant chemoradiotherapy and surgery plus adjuvant chemotherapy; PCT, surgery plus postoperative chemotherapy; PCRT, surgery plus postoperative chemoradiotherapy. [file 12885_2023_11525_MOESM2_ESM.docx]

| Log Rank (Mantel-Cox) | **DFS** | | | |  | | | **OS** | | | |
| --- | --- | --- | --- | --- | --- | --- | --- | --- | --- | --- | --- |
|  | *P* value | | | |  | | | *P* value | | | |
|  | NCRT | Surgery-alone | PCT | PCRT | |  | NCRT | | Surgery-alone | PCT | PCRT |
| NCRT |  | .001 | .228 | .853 | |  |  | | .226 | .256 | .756 |
| Surgery-alone | .001 |  | .017 | .003 | |  | .226 | |  | .883 | .395 |
| PCT | .228 | .017 |  | .317 | |  | .256 | | .883 |  | .425 |
| PCRT | .853 | .003 | .317 |  | |  | .756 | | .395 | .425 |  |
| **Table S2**. Abbreviations: **DFS**, disease-free survival ;**OS**, overall survival; **NCRT**, neoadjuvant chemoradiotherapy and surgery plus adjuvant chemotherapy; **PCT**, surgery plus postoperative chemotherapy; **PCRT**, surgery plus postoperative chemoradiotherapy. | | | | | | | | | | | |
