# Supplementary material for: Survival outcomes in locally advanced dMMR rectal cancer: surgery plus adjunctive treatment vs. surgery alone
Source: BMC Cancer. 2023 Oct 20;23:1013. doi: 10.1186/s12885-023-11525-7 (PMC10588073; doi:10.1186/s12885-023-11525-7)
Supplement: Supplementary file 3 — Additional file 3: Supplementary Fig. 1. (A) Disease-free survival (DFS) of LARC patients treated with surgery-alone and other treatments. (B) Overall survival (OS) of LARC patients treated with surgery alone and other treatments. LARC, locally advanced rectal cancer (stages II and III); HR, hazard ratio; CI, confidence interval. [file 12885_2023_11525_MOESM3_ESM.pdf]

Post-Exclusion of Mucinous Adenocarcinoma

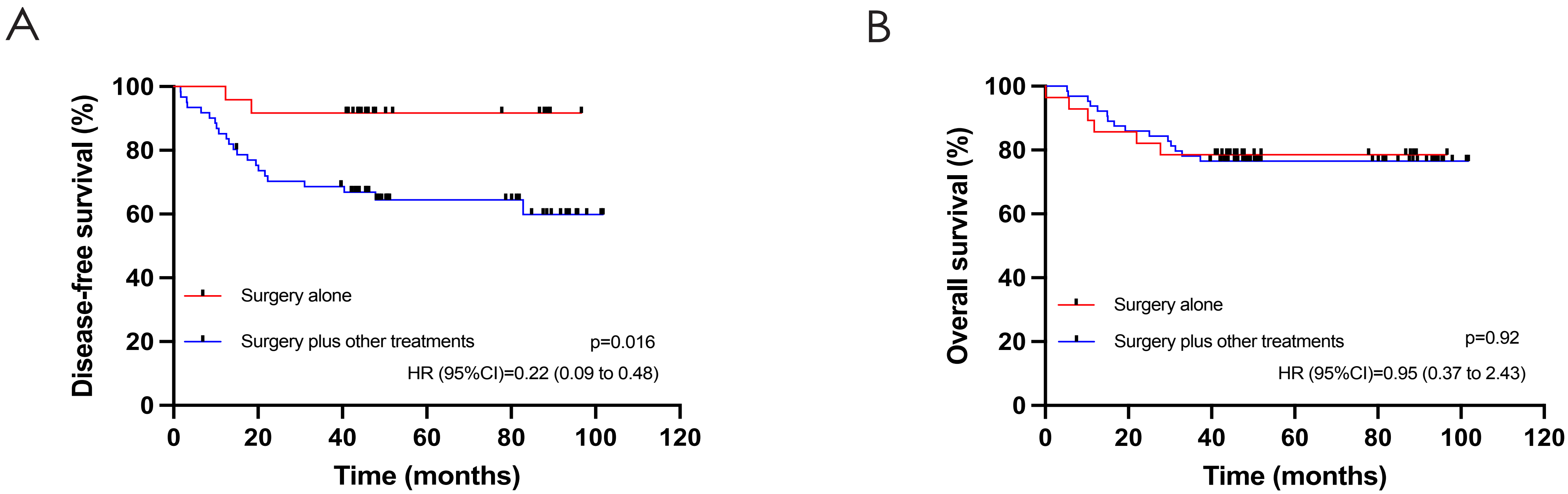

No.at risk

Surgery alone	24	23	23	7	6	1	0
Surgery plus other treatments	61	45	39	19	15	4	0

No.at risk

Surgery alone	28	25	23	7	6	1	0
Surgery plus other treatments	64	56	48	19	17	3	0

Supplementary Fig 1 (A) Disease-free survival (DFS) of LARC patients treated with surgery-alone and other treatments.(B) Overall survival (OS) of LARC patients treated with surgery alone and other treatments.LARC,locally advanced rectal cancer (stages Ⅱ and Ⅲ) ; HR,hazard ratio; CI,confidence interval.
